# (*Z*)-2-(2-Oxoindolin-3-yl­idene)-*N*-phenylhydrazinecarbothio­amide

**DOI:** 10.1107/S160053681200400X

**Published:** 2012-03-07

**Authors:** Amna Qasem Ali, Naser Eltaher Eltayeb, Siang Guan Teoh, Abdussalam Salhin, Hoong-Kun Fun

**Affiliations:** a School of Chemical Sciences, Universiti Sains Malaysia, Minden, Penang, Malaysia; b Faculty of Science, Sabha University, Libya; c Department of Chemistry, International University of Africa, Sudan; d X-ray Crystallography Unit, School of Physics, Universiti Sains Malaysia, 11800 USM, Penang, Malaysia

## Abstract

In the title compound, C_15_H_12_N_4_OS, the dihedral angle between the nine-membered indolin-2-one ring system and the phenyl ring is 2.72 (7)°. Intra­molecular cyclic N—H⋯O and C—H⋯S hydrogen-bonding inter­actions [graph set *S*(6)] are present, as are weak N—H⋯N inter­actions [graph set *S*(5)]. In the crystal, mol­ecules form centrosymmetric cyclic dimers through pairs of N—H⋯O hydrogen bonds [graph set *R*_2_^2^(8)] and these are extended by C—H⋯S inter­actions. The crystal structure also features weak C—H⋯π inter­actions.

## Related literature

For related crystal structures, see: Ali *et al.* (2012 ▶); Qasem Ali *et al.* (2011*a* ▶,*b* ▶); Ferrari *et al.* (2002 ▶); Pervez *et al.* (2010 ▶); Ramzan *et al.* (2010 ▶). For various biological activities of Schiff bases, see: Bhandari *et al.* (2008 ▶); Bhardwaj *et al.* (2010 ▶); Pandeya *et al.* (1999 ▶); Sridhar *et al.* (2002 ▶); Suryavanshi & Pai (2006 ▶). For the cytotoxic and anti­cancer activities of isatin and its derivatives, see: Vine *et al.* (2009 ▶). For graph-set analysis, see Bernstein *et al.* (1995 ▶).
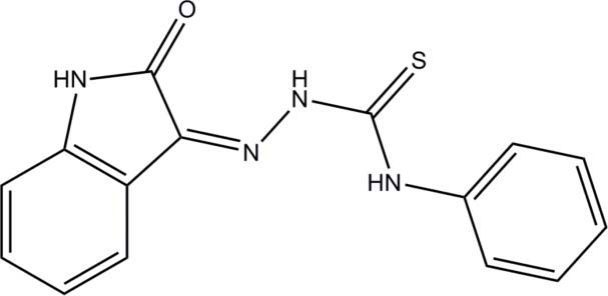


## Experimental

### 

#### Crystal data

C_15_H_12_N_4_OS
*M* *_r_* = 296.35
Monoclinic, 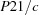

*a* = 6.3674 (1) Å
*b* = 15.4594 (3) Å
*c* = 14.2199 (3) Å
β = 93.383 (1)°
*V* = 1397.31 (5) Å^3^
*Z* = 4
Mo *K*α radiation
μ = 0.24 mm^−1^
*T* = 100 K
0.47 × 0.13 × 0.13 mm

#### Data collection

Bruker APEXII CCD diffractometer
Absorption correction: multi-scan (*SADABS*; Bruker, 2005 ▶) *T*_min_ = 0.897, *T*_max_ = 0.971
15557 measured reflections
4159 independent reflections
2985 reflections with *I* > 2σ(*I*)
*R*_int_ = 0.040

#### Refinement

*R*[*F* ^2^ > 2σ(*F* ^2^)] = 0.053
*wR*(*F* ^2^) = 0.113
*S* = 1.02
4159 reflections
202 parameters
H atoms treated by a mixture of independent and constrained refinement
Δρ_max_ = 0.34 e Å^−3^
Δρ_min_ = −0.49 e Å^−3^

### 

Data collection: *APEX2* (Bruker, 2005 ▶); cell refinement: *SAINT* (Bruker, 2005 ▶); data reduction: *SAINT*; program(s) used to solve structure: *SHELXS97* (Sheldrick, 2008 ▶); program(s) used to refine structure: *SHELXL97* (Sheldrick, 2008 ▶); molecular graphics: *SHELXTL* (Sheldrick, 2008 ▶); software used to prepare material for publication: *SHELXTL* and *PLATON* (Spek, 2009 ▶).

## Supplementary Material

Crystal structure: contains datablock(s) I, global. DOI: 10.1107/S160053681200400X/wn2464sup1.cif

Structure factors: contains datablock(s) I. DOI: 10.1107/S160053681200400X/wn2464Isup2.hkl

Supplementary material file. DOI: 10.1107/S160053681200400X/wn2464Isup3.cml

Additional supplementary materials:  crystallographic information; 3D view; checkCIF report

## Figures and Tables

**Table 1:** Hydrogen-bond geometry (Å, °) *Cg*2 is the centroid of the C1–C6 ring and *Cg*3 is the centroid of the C10–C15 ring.

*D*—H⋯*A*	*D*—H	H⋯*A*	*D*⋯*A*	*D*—H⋯*A*
N1—H1N1⋯O1^i^	0.88 (2)	2.00 (2)	2.8737 (18)	173.9 (18)
N3—H1N3⋯O1	0.87 (2)	2.07 (2)	2.7646 (18)	136 (2)
N4—H1N4⋯N2	0.88 (2)	2.05 (2)	2.5781 (19)	117.4 (17)
C11—H11*A*⋯S1^ii^	0.95	2.83	3.6017 (19)	139
C15—H15*A*⋯S1	0.95	2.60	3.2735 (19)	128
C2—H2*A*⋯*Cg*3^iii^	0.95	2.80	3.510 (2)	132
C13—H13*A*⋯*Cg*2^iv^	0.95	2.82	3.5201 (19)	131

Symmetry codes: (i) 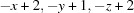; (ii) 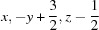; (iii) 
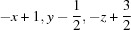
; (iv) 
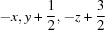
.

**Fig. 1.**
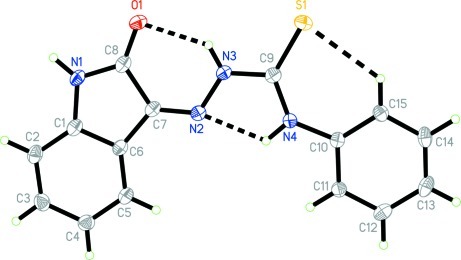
The molecular structure of the title compound, with 50% probability displacement ellipsoids. Dashed lines indicate hydrogen bonds.

**Fig. 2.**
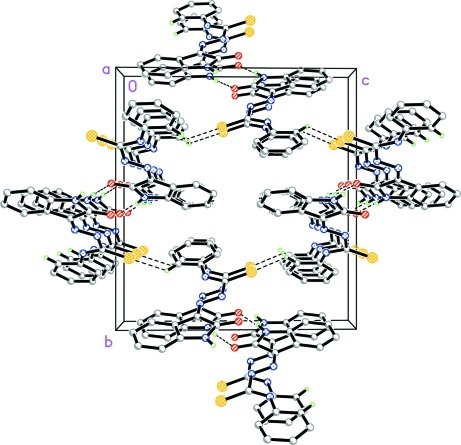
The crystal packing of the title compound, viewed down the a axis. Intermolecular hydrogen bonds are shown as dashed lines. H atoms not involved in the hydrogen bond interactions have been omitted for clarity.

## supplementary crystallographic information

### Comment

Isatin (2,3-dioxindole) is an endogenous compound identified in humans, and its effect has been studied in a variety of systems. The biological properties of isatin and its derivatives include a range of actions in the brain, offer protection against bacterial (Suryavanshi & Pai, 2006) and fungal infections and possess anticonvulsant, anti-HIV (Pandeya *et al.*, 1999), antidepressant and anti-inflammatory activities (Bhandari *et al.*, 2008). Recently, we reported the crystal structure of (*Z*)-2-(5-chloro-2-oxoindolin-3-ylidene)-*N*-phenylhydrazinecarbothioamide (Qasem Ali *et al.*, 2011*a*). In the present paper we describe the single-crystal X-ray diffraction study of the title compound (Fig. 1).

In the title compound, C_15_H_12_N_4_OS, the dihedral angle between the nine-membered indolin-2-one ring system and the phenyl ring is 2.72 (7)°. These two ring systems are connected by a chain of four atoms N2—N3—C9—N4; this torsion angle is 4.1 (2)°. The torsion angles C7—N2—N3—C9 and C10—N4—C9—N3 are 173.69 (15)° and 174.00 (16)°, respectively. These values are very close to those in a similar structure (Qasem Ali *et al.*, 2011*a*).

The essentially planar conformation of the molecule is maintained by cyclic intramolecular N3—H1N3···O1 and C15—H15A···S1 hydrogen-bonding interactions [graph set *S*(6) (Bernstein *et al.*, 1995)] (Table 1), together with a weak *S*(5) N4—H1N4···N2 interaction.

In the crystal structure, the molecules form centrosymmetric cyclic dimers through intermolecular N1—H1N1···O1 hydrogen bonds [graph set *R*^2^_2_(8)] and are extended by C11—H11A···S1 hydrogen bond interactions.

The crystal structure (Fig. 2) is stabilized by weak C—H···π interactions (Table 1) involving the C10–C15 ring (centroid Cg3) and C1–C6 ring (centroid Cg2).

### Experimental

The Schiff base has been synthesized by refluxing the reaction mixture of a hot ethanolic solution (30 ml) of 4-phenyl-3-thiosemicarbazide (0.01 mol) and a hot ethanolic solution (30 ml) of isatin (0.01 mol) for 2 h. The precipitate formed during reflux was filtered, washed with cold EtOH and recrystallized from hot EtOH. Yield (m.p.): 90% (510.2–511.6 K). The yellow crystals were grown in acetone–dimethylformamide (3:1) by slow evaporation at room temperature.

### Refinement

N-bound H atoms were located in a difference Fourier map and were refined freely; N—H = 0.87 (2) Å and 0.88 (2) Å. The remaining H atoms were positioned geometrically and refined using a riding model, with C—H = 0.95 Å and *U*_iso_(H) = 1.2*U*_eq_(C).

### Crystal data

**Table:**

C_15_H_12_N_4_OS	*F*(000) = 616
*M**_r_* = 296.35	*D*_x_ = 1.409 Mg m^−^^3^
Monoclinic, *P*2_1_/*c*	Melting point = 510.2–511.6 K
Hall symbol: -P 2ybc	Mo *K*α radiation, λ = 0.71073 Å
*a* = 6.3674 (1) Å	Cell parameters from 4743 reflections
*b* = 15.4594 (3) Å	θ = 3.0–30.1°
*c* = 14.2199 (3) Å	µ = 0.24 mm^−^^1^
β = 93.383 (1)°	*T* = 100 K
*V* = 1397.31 (5) Å^3^	Needle, yellow
*Z* = 4	0.47 × 0.13 × 0.13 mm

### Data collection

**Table:**

Bruker APEXII CCD diffractometer	4159 independent reflections
Radiation source: fine-focus sealed tube	2985 reflections with *I* > 2σ(*I*)
Graphite monochromator	*R*_int_ = 0.040
φ and ω scans	θ_max_ = 30.3°, θ_min_ = 2.0°
Absorption correction: multi-scan (*SADABS*; Bruker, 2005)	*h* = −9→8
*T*_min_ = 0.897, *T*_max_ = 0.971	*k* = −21→18
15557 measured reflections	*l* = −17→20

### Refinement

**Table:**

Refinement on *F*^2^	Primary atom site location: structure-invariant direct methods
Least-squares matrix: full	Secondary atom site location: difference Fourier map
*R*[*F*^2^ > 2σ(*F*^2^)] = 0.053	Hydrogen site location: inferred from neighbouring sites
*wR*(*F*^2^) = 0.113	H atoms treated by a mixture of independent and constrained refinement
*S* = 1.02	*w* = 1/[σ^2^(*F*_o_^2^) + (0.038*P*)^2^ + 0.7736*P*] where *P* = (*F*_o_^2^ + 2*F*_c_^2^)/3
4159 reflections	(Δ/σ)_max_ = 0.001
202 parameters	Δρ_max_ = 0.34 e Å^−^^3^
0 restraints	Δρ_min_ = −0.49 e Å^−^^3^

### Special details

**Table:**

Geometry. All e.s.d.'s (except the e.s.d. in the dihedral angle between two l.s. planes) are estimated using the full covariance matrix. The cell e.s.d.'s are taken into account individually in the estimation of e.s.d.'s in distances, angles and torsion angles; correlations between e.s.d.'s in cell parameters are only used when they are defined by crystal symmetry. An approximate (isotropic) treatment of cell e.s.d.'s is used for estimating e.s.d.'s involving l.s. planes.
Refinement. Refinement of *F*^2^ against ALL reflections. The weighted *R*-factor *wR* and goodness of fit *S* are based on *F*^2^, conventional *R*-factors *R* are based on *F*, with *F* set to zero for negative *F*^2^. The threshold expression of *F*^2^ > σ(*F*^2^) is used only for calculating *R*-factors(gt) *etc*. and is not relevant to the choice of reflections for refinement. *R*-factors based on *F*^2^ are statistically about twice as large as those based on *F*, and *R*-factors based on ALL data will be even larger.

### Fractional atomic coordinates and isotropic or equivalent isotropic displacement parameters (Å^2^)

**Table:**

	*x*	*y*	*z*	*U*_iso_*/*U*_eq_	
S1	0.15701 (8)	0.72368 (4)	1.06040 (3)	0.03145 (15)	
O1	0.73491 (19)	0.55698 (8)	1.00760 (8)	0.0228 (3)	
N1	0.9075 (2)	0.50533 (10)	0.87950 (10)	0.0208 (3)	
N2	0.4169 (2)	0.61290 (9)	0.85318 (10)	0.0177 (3)	
N3	0.3689 (2)	0.63773 (9)	0.94027 (10)	0.0191 (3)	
N4	0.0843 (2)	0.70518 (9)	0.87089 (10)	0.0181 (3)	
C1	0.8690 (3)	0.50379 (11)	0.78102 (12)	0.0195 (4)	
C2	0.9950 (3)	0.47108 (12)	0.71370 (13)	0.0241 (4)	
H2A	1.1264	0.4445	0.7306	0.029*	
C3	0.9210 (3)	0.47880 (12)	0.61995 (13)	0.0253 (4)	
H3A	1.0027	0.4560	0.5719	0.030*	
C4	0.7296 (3)	0.51916 (12)	0.59478 (13)	0.0231 (4)	
H4A	0.6846	0.5243	0.5301	0.028*	
C5	0.6042 (3)	0.55185 (11)	0.66319 (12)	0.0206 (4)	
H5A	0.4741	0.5794	0.6462	0.025*	
C6	0.6740 (3)	0.54324 (11)	0.75712 (12)	0.0184 (4)	
C7	0.5904 (3)	0.57114 (11)	0.84497 (12)	0.0176 (3)	
C8	0.7491 (3)	0.54476 (11)	0.92218 (12)	0.0188 (4)	
C9	0.1964 (3)	0.68962 (11)	0.95214 (12)	0.0185 (4)	
C10	−0.1096 (3)	0.74787 (11)	0.85012 (12)	0.0168 (3)	
C11	−0.1753 (3)	0.74938 (11)	0.75467 (12)	0.0190 (4)	
H11A	−0.0892	0.7245	0.7095	0.023*	
C12	−0.3656 (3)	0.78705 (11)	0.72568 (13)	0.0237 (4)	
H12A	−0.4098	0.7878	0.6607	0.028*	
C13	−0.4917 (3)	0.82369 (11)	0.79112 (13)	0.0247 (4)	
H13A	−0.6228	0.8492	0.7714	0.030*	
C14	−0.4248 (3)	0.82279 (11)	0.88559 (13)	0.0229 (4)	
H14A	−0.5106	0.8486	0.9303	0.027*	
C15	−0.2349 (3)	0.78504 (11)	0.91642 (12)	0.0196 (4)	
H15A	−0.1912	0.7846	0.9815	0.024*	
H1N1	1.018 (3)	0.4836 (14)	0.9106 (15)	0.037 (6)*	
H1N3	0.457 (4)	0.6258 (14)	0.9879 (16)	0.039 (6)*	
H1N4	0.144 (3)	0.6806 (14)	0.8234 (15)	0.029 (6)*	

### Atomic displacement parameters (Å^2^)

**Table:**

	*U*^11^	*U*^22^	*U*^33^	*U*^12^	*U*^13^	*U*^23^
S1	0.0267 (2)	0.0507 (3)	0.0168 (2)	0.0076 (2)	0.00014 (18)	−0.0045 (2)
O1	0.0242 (6)	0.0248 (7)	0.0189 (6)	0.0016 (5)	−0.0032 (5)	0.0026 (5)
N1	0.0191 (7)	0.0199 (8)	0.0228 (8)	0.0043 (6)	−0.0032 (6)	0.0020 (6)
N2	0.0189 (7)	0.0153 (7)	0.0187 (7)	−0.0008 (6)	0.0001 (5)	0.0008 (6)
N3	0.0184 (7)	0.0216 (8)	0.0171 (7)	0.0022 (6)	−0.0011 (6)	0.0020 (6)
N4	0.0184 (7)	0.0193 (8)	0.0166 (7)	0.0032 (6)	0.0014 (6)	0.0000 (6)
C1	0.0195 (8)	0.0131 (8)	0.0254 (9)	0.0000 (7)	−0.0025 (7)	0.0014 (7)
C2	0.0211 (9)	0.0197 (9)	0.0315 (10)	0.0045 (7)	0.0006 (8)	0.0030 (8)
C3	0.0265 (9)	0.0231 (9)	0.0268 (9)	0.0049 (8)	0.0064 (8)	0.0016 (8)
C4	0.0248 (9)	0.0220 (9)	0.0225 (9)	0.0017 (7)	0.0004 (7)	0.0018 (7)
C5	0.0187 (8)	0.0181 (9)	0.0248 (9)	0.0010 (7)	−0.0016 (7)	0.0016 (7)
C6	0.0190 (8)	0.0140 (8)	0.0220 (8)	0.0005 (7)	−0.0007 (7)	0.0009 (7)
C7	0.0183 (8)	0.0127 (8)	0.0213 (8)	−0.0015 (6)	−0.0020 (7)	0.0023 (6)
C8	0.0187 (8)	0.0120 (8)	0.0254 (9)	−0.0013 (7)	−0.0021 (7)	0.0031 (7)
C9	0.0173 (8)	0.0186 (8)	0.0195 (8)	−0.0036 (7)	0.0016 (6)	0.0035 (7)
C10	0.0164 (8)	0.0136 (8)	0.0205 (8)	−0.0016 (6)	0.0017 (6)	0.0014 (6)
C11	0.0198 (8)	0.0179 (8)	0.0194 (8)	0.0002 (7)	0.0023 (7)	0.0013 (7)
C12	0.0243 (9)	0.0204 (9)	0.0257 (9)	−0.0013 (7)	−0.0043 (7)	0.0044 (7)
C13	0.0187 (8)	0.0167 (9)	0.0383 (11)	0.0006 (7)	−0.0014 (8)	0.0044 (8)
C14	0.0201 (8)	0.0153 (8)	0.0339 (10)	0.0008 (7)	0.0066 (7)	−0.0026 (8)
C15	0.0214 (8)	0.0165 (8)	0.0211 (8)	−0.0007 (7)	0.0024 (7)	−0.0021 (7)

### Geometric parameters (Å, º)

**Table:**

S1—C9	1.6600 (18)	C4—C5	1.389 (3)
O1—C8	1.238 (2)	C4—H4A	0.9500
N1—C8	1.352 (2)	C5—C6	1.389 (2)
N1—C1	1.408 (2)	C5—H5A	0.9500
N1—H1N1	0.88 (2)	C6—C7	1.452 (2)
N2—C7	1.291 (2)	C7—C8	1.504 (2)
N2—N3	1.349 (2)	C10—C15	1.394 (2)
N3—C9	1.378 (2)	C10—C11	1.397 (2)
N3—H1N3	0.87 (2)	C11—C12	1.385 (2)
N4—C9	1.343 (2)	C11—H11A	0.9500
N4—C10	1.416 (2)	C12—C13	1.386 (3)
N4—H1N4	0.88 (2)	C12—H12A	0.9500
C1—C2	1.381 (3)	C13—C14	1.385 (3)
C1—C6	1.407 (2)	C13—H13A	0.9500
C2—C3	1.393 (3)	C14—C15	1.390 (2)
C2—H2A	0.9500	C14—H14A	0.9500
C3—C4	1.397 (2)	C15—H15A	0.9500
C3—H3A	0.9500		
			
C8—N1—C1	111.31 (14)	N2—C7—C6	125.83 (15)
C8—N1—H1N1	123.0 (14)	N2—C7—C8	127.70 (16)
C1—N1—H1N1	125.7 (14)	C6—C7—C8	106.43 (14)
C7—N2—N3	117.83 (14)	O1—C8—N1	127.51 (15)
N2—N3—C9	120.23 (14)	O1—C8—C7	126.16 (16)
N2—N3—H1N3	118.7 (15)	N1—C8—C7	106.34 (15)
C9—N3—H1N3	120.6 (15)	N4—C9—N3	112.68 (15)
C9—N4—C10	132.49 (16)	N4—C9—S1	129.71 (14)
C9—N4—H1N4	110.5 (13)	N3—C9—S1	117.61 (12)
C10—N4—H1N4	116.9 (13)	C15—C10—C11	119.98 (15)
C2—C1—C6	122.14 (16)	C15—C10—N4	125.29 (15)
C2—C1—N1	128.43 (15)	C11—C10—N4	114.71 (15)
C6—C1—N1	109.42 (15)	C12—C11—C10	120.19 (17)
C1—C2—C3	117.03 (16)	C12—C11—H11A	119.9
C1—C2—H2A	121.5	C10—C11—H11A	119.9
C3—C2—H2A	121.5	C11—C12—C13	120.24 (16)
C2—C3—C4	121.63 (18)	C11—C12—H12A	119.9
C2—C3—H3A	119.2	C13—C12—H12A	119.9
C4—C3—H3A	119.2	C14—C13—C12	119.32 (16)
C5—C4—C3	120.78 (16)	C14—C13—H13A	120.3
C5—C4—H4A	119.6	C12—C13—H13A	120.3
C3—C4—H4A	119.6	C13—C14—C15	121.48 (17)
C6—C5—C4	118.29 (16)	C13—C14—H14A	119.3
C6—C5—H5A	120.9	C15—C14—H14A	119.3
C4—C5—H5A	120.9	C14—C15—C10	118.79 (16)
C5—C6—C1	120.11 (17)	C14—C15—H15A	120.6
C5—C6—C7	133.33 (16)	C10—C15—H15A	120.6
C1—C6—C7	106.50 (14)		
			
C7—N2—N3—C9	173.69 (15)	C1—N1—C8—O1	−179.73 (17)
C8—N1—C1—C2	178.50 (18)	C1—N1—C8—C7	0.46 (18)
C8—N1—C1—C6	−0.7 (2)	N2—C7—C8—O1	2.3 (3)
C6—C1—C2—C3	0.1 (3)	C6—C7—C8—O1	−179.84 (16)
N1—C1—C2—C3	−179.09 (17)	N2—C7—C8—N1	−177.88 (17)
C1—C2—C3—C4	1.1 (3)	C6—C7—C8—N1	−0.03 (18)
C2—C3—C4—C5	−1.1 (3)	C10—N4—C9—N3	174.00 (16)
C3—C4—C5—C6	−0.1 (3)	C10—N4—C9—S1	−6.4 (3)
C4—C5—C6—C1	1.2 (3)	N2—N3—C9—N4	4.1 (2)
C4—C5—C6—C7	177.76 (18)	N2—N3—C9—S1	−175.54 (12)
C2—C1—C6—C5	−1.3 (3)	C9—N4—C10—C15	−0.6 (3)
N1—C1—C6—C5	178.05 (15)	C9—N4—C10—C11	−179.21 (17)
C2—C1—C6—C7	−178.61 (16)	C15—C10—C11—C12	−0.6 (3)
N1—C1—C6—C7	0.69 (19)	N4—C10—C11—C12	178.11 (15)
N3—N2—C7—C6	−176.52 (15)	C10—C11—C12—C13	0.2 (3)
N3—N2—C7—C8	0.9 (3)	C11—C12—C13—C14	0.5 (3)
C5—C6—C7—N2	0.6 (3)	C12—C13—C14—C15	−0.8 (3)
C1—C6—C7—N2	177.50 (16)	C13—C14—C15—C10	0.4 (3)
C5—C6—C7—C8	−177.26 (19)	C11—C10—C15—C14	0.3 (2)
C1—C6—C7—C8	−0.40 (18)	N4—C10—C15—C14	−178.24 (16)

### Hydrogen-bond geometry (Å, º)

Cg2 is the centroid of the C1–C6 ring and Cg3 is the centroid of the C10–C15 ring.

**Table:**

*D*—H···*A*	*D*—H	H···*A*	*D*···*A*	*D*—H···*A*
N1—H1*N*1···O1^i^	0.88 (2)	2.00 (2)	2.8737 (18)	173.9 (18)
N3—H1*N*3···O1	0.87 (2)	2.07 (2)	2.7646 (18)	136 (2)
N4—H1*N*4···N2	0.88 (2)	2.05 (2)	2.5781 (19)	117.4 (17)
C11—H11*A*···S1^ii^	0.95	2.83	3.6017 (19)	139
C15—H15*A*···S1	0.95	2.60	3.2735 (19)	128
C2—H2*A*···*Cg*3^iii^	0.95	2.80	3.510 (2)	132
C13—H13*A*···*Cg*2^iv^	0.95	2.82	3.5201 (19)	131

Symmetry codes: (i) −*x*+2, −*y*+1, −*z*+2; (ii) *x*, −*y*+3/2, *z*−1/2; (iii) −*x*+1, *y*−1/2, −*z*+3/2; (iv) −*x*, *y*+1/2, −*z*+3/2.
